# O3.8. DORSOLATERAL PREFRONTAL CORTEX IN DRUG-NAïVE FIRST EPISODE SCHIZOPHRENIA: DYNAMIC PHASE COHERENCE OF INFRASLOW OSCILLATIONS

**DOI:** 10.1093/schbul/sby015.206

**Published:** 2018-04-01

**Authors:** Tushar Das, Mingli Li, Lena Palaniyappan, Tao Li

**Affiliations:** 1 University of Western Ontario; 2 Mental Health Center, West China Hospital of Sichuan University

## Abstract

**Background:**

Dorsolateral Prefrontal Cortex (DLPFC) has been identified as the seat of many synaptic changes in schizophrenia. Functional neuroimaging studies indicate inefficient recruitment of DLPFC, often termed as hypofrontality, that appears in conjunction with disrupted connectivity of DLPFC with other brain regions. Very Low Frequency Oscillations (VLFO or infraslow oscillations) stem from AMPA currents, and are thought to represent a slow, cyclic modulation of cortical gross excitability. These oscillations are phase-synchronised enabling long-distance communication. This synchrony fluctuates across time (dynamic), indicating state-shifts. Dynamic synchrony can be captured using variance of phase coherence (vPC). We aimed to isolate the brain regions showing abnormal vPC with right DLPFC in drug-naïve first episode schizophrenia compared to healthy controls. Based on our prior work indicating fronto-insular dysconnectivity, we hypothesized that anterior insula would show the most disrupted dynamic phase coherence with DLPFC among all other brain regions.

**Methods:**

129 drug-naïve patients with first episode of schizophrenia (FES) and 197 age- sex- and education-level matched healthy controls (HC) were recruited. Based on Dosenbach’s atlas applied to 7.67 minutes (230 timepoints with TR=2 s) of eyes-open resting fMRI scan, we extracted timeseries of 160 functional network nodes, and identified right DLPFC with MNI coordinates (x=40,y=36,z=29). Wavelet-transformation was done to enable time-frequency analysis that decomposed the timeseries data into 3 bins of very low frequency oscillations (0.02–0.04 Hz, 0.04–0.06 Hz, 0.06–0.08 Hz). We then estimated phase coherence between DLPFC and other 159 regions at each timepoint, and the variance (vPC) across the entire acquisition. Regions showing aberrant vPC with right DLPFC were identified using FDR corrected 2-tailed p<0.05 as the threshold of statistical significance in a two-sample t test comparing HC and FES groups. A two-step clustering procedure using likelihood-distance measure was employed to stratify sub-groups of FES with or without vPC disruption. The resulting subgroups were compared based on clinical symptom scores using van der Gaag’s 5-factor PANSS model.

**Results:**

The FES group showed a significant reduction (FDR corrected p = 0.03) in vPC between right DLPFC and left anterior insula within the frequency band: 0.02 - 0.04 Hz. The rDLPFC-lAI path can be termed as a long-distance connection based on anatomical distance measure (82.6 mm, compared to a median of 75mm) when compared to HCs. FES group was split into 2 subgroups using the clustering procedure. FES-2 (n=51) had higher vPC of rDLPFC-lAI connectivity compared to HC (Hedges’ g: -2.0, p=0.001) while FES-1 (n=78) had lower vPC compared to HC (Hedges’ g: 0.47, p=0.009). FES-1 had more ‘emotional’ and ‘positive symptoms’ but had similar symptom loadings in other PANSS domains compared to FES-2.

**Discussion:**

Reduced variability of phase coherence between anterior insula and DLPFC in schizophrenia confirms our prior hypothesis of Salience Executive Loop dysfunction in this illness. Current results indicate that aberrant connectivity with anterior insula is the major lateral prefrontal disruption even in a drug naïve state at rest. Further, this aberration seems to be specific to infraslow rather than other frequency bands, raising the possibility that this may be a key mechanism of modulating overall cortical excitability in schizophrenia. It is possible that this dysfunction is specific to a subgroup with more psychotic symptoms at first presentation.

